# Passive Source Localization Using Acoustic Intensity in Multipath-Dominant Shallow-Water Waveguide

**DOI:** 10.3390/s21062198

**Published:** 2021-03-21

**Authors:** Sunhyo Kim, Sungho Cho, Seom-kyu Jung, Jee Woong Choi

**Affiliations:** 1 Marine Security and Safety Research Center, Korea Institute of Ocean Science and Technology, Busan 49111, Korea; sunhyo@kiost.ac.kr (S.K.); shcho@kiost.ac.kr (S.C.); skjung@kiost.ac.kr (S.-k.J.); 2 Department of Marine Science and Convergence Engineering, Hanyang University-ERICA, Ansan, Gyeonggido 15588, Korea

**Keywords:** particle velocity, array invariant, passive source localization

## Abstract

The array invariant technique has been recently proposed for passive source localization in the ocean. It has successfully estimated the source–receiver horizontal range in multipath-dominant shallow-water waveguides. However, it requires a relatively large-scale hydrophone array. This study proposes an array invariant method that uses acoustic intensity, which is a vector quantity that has the same direction as the sound wave propagating through a water medium. This method can be used to estimate not only the source–receiver horizontal range, but also the azimuth to an acoustic source. The feasibility of using a vector quantity for the array invariant method is examined through a simulation and an acoustic experiment in which particle velocity signals are obtained using a finite difference approximation of the pressure signals at two adjacent points. The source localization results estimated using acoustic intensity are compared with those obtained from beamforming of the acoustic signals acquired by the vertical line array.

## 1. Introduction

Acoustic source localization in ocean waveguides using vertical or horizontal receiver arrays has been studied in past decades. Representative localization techniques include matched-field processing [1,2,3] and the array invariant technique [4,5,6,7,8,9,10]. Matched-field processing is a method of estimating the source location as a function of range and depth by analyzing the correlation between replicas predicted by an acoustic propagation model and the acoustic pressure field received by a vertical receiver array [1]. Therefore, this method requires accurate knowledge on the acoustic propagation environment (e.g., sound speed profile and sediment geoacoustic parameters) and receiver geometry (e.g., array tilt and position) [2,3].

The array invariant technique was proposed for robust source localization in recent years. Since sound propagation in shallow water is greatly influenced by multiple interactions of sound with ocean boundaries such as the sea surface and seabed, sound propagating through multipaths is sequentially received by a hydrophone with different arrival times. The array invariant uses the beam-time migration (separated by beam angle and arrival time) of multipaths in shallow water [4,5,6,7]. An advantage of the array invariant technique is that it does not require detailed knowledge on the shallow water environment and excessive computational effort, and it is a robust method for multipath-dominant ocean waveguides. The array invariant technique has been successfully applied to source–range estimation for surface and submerged sources in shallow water environments [6,7,8,9,10]. However, it requires a relatively large-scale receiver array because the beam-time migration is derived via beamforming using a vertical or horizontal receiver array.

In this study, the beam-time migration curve is derived using acoustic intensity. Acoustic intensity is a vector quantity with three directional components at a single point in space. Therefore, it can be utilized to estimate passive source localization via the array invariant technique while maximizing spatial efficiency.

This paper is organized as follows. Section 2 provides a description of the array invariant technique using the x, y, and z components of acoustic intensity. Section 3 presents the simulation results of source localization at various source–receiver distances and directions. Section 4 describes the acoustic experiment conducted on a multipath-dominant shallow-water waveguide. The passive source locations are evaluated from signals measured from two adjacent hydrophones during this experiment, and the results are compared with those obtained from beamforming of the acoustic signals acquired by the vertical line array in Section 5. The summary and conclusions are provided in Section 6.

## 2. Passive Source Localization Using Acoustic Intensity Vector

### 2.1. Array Invariant

The waveguide invariant (denoted β) is defined as the ratio of the change of modal group slowness to the change of phase slowness, it represents the fluctuations in acoustic intensity field with frequency and distance from the acoustic source to the receiver [11,12,13,14]. The array invariant (denoted χ) proposed by Lee and Makris [4] is a method that estimates instantaneous source range in a horizontally stratified ocean waveguide using the beam-time intensity data obtained by conventional plane-wave beamforming. Song and Cho [7] recently showed that when β is 1, the array invariant corresponds to a special case of waveguide invariant. In addition, they demonstrated that if only low-order modes are utilized for range estimation, the array invariant can be extended to the cases where β is not 1.

In general, the array invariant method is useful in a multipath-dominant shallow-water waveguide. In this case, the array invariant parameter χ is defined as

(1) $$\chi \equiv \;\frac{d}{dt}\;\left(\cos\theta \right)=\;\frac{d}{dt}\sqrt{1-s^{2}},$$

where θ is the arrival angle of each multipath, which is the angle between the horizontal axis and the acoustic beam; t is the travel time of each arrival; and s is sinθ. Equation (1) leads to an elliptic curve form whose major and minor axis coincide with the beam-time (s, t) coordinate, as follows:

(2) $${\left(\frac{t-t_{1}}{1/\chi }\right)}^{2}+\sin^{2}\theta =1.$$

The center of the elliptic curve is located on the t-axis at ($\theta_{1},t_{1}$), where the subscript 1 means the first arrival, and 1/|χ| is the horizontal semimajor axis. The array invariant parameter χ can be numerically estimated from beam-time migration data by using the least-squares (LS) approach [4,5,6,7] in the multipath-dominant shallow-water waveguide. Integrating Equation (1) over time is expressed as a linear equation with a slope of χ and a y-intercept constant C, as follows:

(3) $$\sqrt{1-s^{2}}=\chi t+C.$$

The best estimate of χ can be obtained by finding the LS solution between the model predictions and measured values obtained from the arrival data separated by beam angle and travel time in the beam-time (s, t) coordinate, as follows [4,5,6,7]:

(4) $${\left[\hat{\chi }\;\hat{C}\right]}^{T}=\;{\left(T^{T}T\right)}^{-1}T^{T}S,$$

where the hat operator means the estimated value obtained by the LS approach. $T=\;\left[{\left(t_{1},t_{2},\ldots ,\;t_{N}\;\right)}^{T}\;1^{T}\right]$, $S=\;{\left\{{\left[1-s_{max}^{2}\left(t_{1}\right)\right]}^{1/2},\;{\left[1-s_{max}^{2}\left(t_{2}\right)\right]}^{1/2},\;\ldots ,\;{\left[1-s_{max}^{2}\left(t_{N}\right)\right]}^{1/2}\right\}}^{T}$, where 1 is a 1×N matrix given by 1 = [1, 1, …, 1], and $s_{max}$ is the maximum peak in beam-time (s, t) coordinates. Then, the horizontal range $\hat{r_{0}}$ between the source and receiver can be estimated simply as [5,6]

(5) $${\hat{r}}_{0}=-\beta \left(\frac{c}{\hat{\chi }}\right),$$

where c is the speed of sound at the receiver.

**Figure 1:**
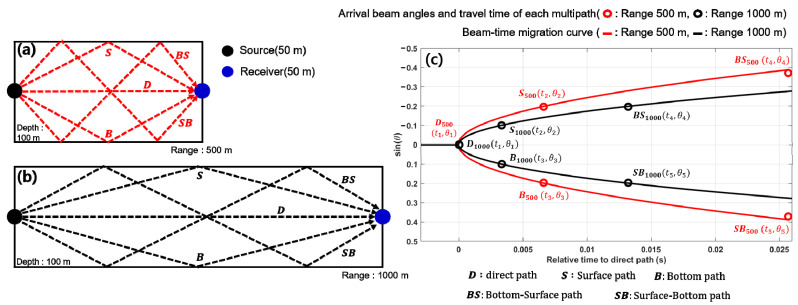
Eigenray outputs for source–receiver ranges of (**a**) 500 m and (**b**) 1000 m, and (**c**) beam-time migration curves for the multipaths of source-receiver ranges 500 m and 1000 m.

Figure 1 is an example illustrating the concept of source–receiver range estimation using the array invariant method in a shallow-water waveguide of 100-m depth with an isovelocity profile. Figure 1a,b show eigenrays corresponding to the first five arrivals consisting of direct, surface, bottom, surface–bottom, and bottom–surface paths for source–receiver ranges of 500 and 1000 m, respectively. Figure 1c shows the distributions of beam-time migration for each eigenray and their best-fit elliptic curves. From the simulation results, it can be seen that the eigenrays for different source–receiver ranges produce the beam-time migration distributions corresponding to elliptic curves with different widths.

The waveguide invariant factor β corresponds to $cos^{2}\theta$ ($=1-s^{2}$) for an isovelocity waveguide. Therefore, for low-order modes with small arrival angles, β converges to unity. Song and Cho [7] showed that the beam-time migration curve is almost identical to the ellipse curve using β=1 for |s|<0.4. In previous studies, beam-time migration was derived via beamforming of signals received by the receiver array and the arrival times of the multipaths were estimated through a complex process, such as finding peaks that exceeded the threshold, clustering the peaks, and identifying the maximum peak in each cluster [7]. However, the estimate of the array invariant parameter may include errors during this complicated process, causing performance degradation of the passive source localization system.

### 2.2. Beam-Time Migration Using Acoustic Intensity

Recently, several studies have been conducted to apply acoustic vector quantities, such as acoustic particle velocity, acceleration, and displacement, which are measured by an acoustic vector sensor. As vector quantities have direction as well as magnitude, a vector sensor has the capability to accurately estimate the azimuth and elevation angles of a submerged source while avoiding left–right ambiguity, which is a problem of the line array receiver consisting of hydrophones. Thus, vector sensors have been applied in several areas, including underwater target tracking, acoustic noise reduction, and underwater communication [15,16,17,18,19,20,21,22,23].

Acoustic particle velocity v(t) can be calculated by Euler’s equation with respect to time [18,19,20,21,22,23]. If two pressure sensors are close together, the pressure gradient along the axis of two pressure sensors can be approximated through finite difference approximation. Then, v(t) is obtained by time integration of the pressure gradient:

(6) $$v\left(t\right)=\frac{1}{\rho_{0}}\int_{0}^{t}\frac{p_{m2}\left(\tau \right)-p_{m1}\left(\tau \right)}{d}d\tau ,$$

where $p_{m1}$ and $p_{m2}$ are the acoustic pressures measured at two adjacent receivers, d is the distance between two receivers, τ is the time variable, and $\rho_{0}$ is the water density. The complex sound intensity describes the instantaneous propagating and nonpropagating energy flux through the medium. Complex sound intensity $I_{c}$ is defined by the product of complex sound pressure p and complex particle velocity v as follows [18,21,22,23]:

(7) $$I_{c}\left(t\right)=\frac{p\left(t\right)v^{*}\left(t\right)}{2},$$

where the asterisk denotes the complex conjugate. Complex representations of acoustic pressure and particle velocity consist of measured real-valued pressure and particle velocity signals and their phase-quadrature signals obtained by the Hilbert transform of the real-valued signals. The real and imaginary parts of $I_{c}$ correspond to the envelopes of active and reactive intensities, respectively, which represent propagating and nonpropagating energy fluxes [18,21,22,23]. If the multipaths do not overlap each other when a far-field condition (kr≫1, where k is the acoustic wavenumber and r is the range from the source) is satisfied, the active intensity is much larger than the reactive intensity. In this case, the direction of active intensity is the same as the propagation direction of the acoustic wave, and the arrival angle of each multipath can be easily estimated by $\theta_{i}=\;\tan^{-1}\left(I_{h,i}/I_{v,i}\right)$, where $\theta_{i}$ is the arrival angle of the i-th multipath. The subscripts h and v indicate the horizontal and vertical components of active intensity, respectively. Parameter χ can be evaluated from the LS solution between the predictions of Equations (3) and (4) and the data obtained using the measured beam angles of multipaths as a function of travel time.

## 3. Numerical Simulation

**Figure 2:**
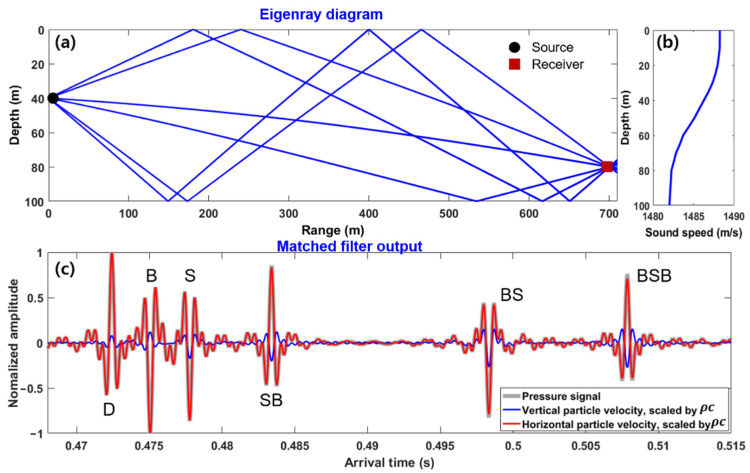
(**a**) Six eigenrays: direct (D), bottom (B), sea surface (S), surface–bottom (S–B), bottom–surface (B–S), and bottom–surface–bottom (B–S–B) paths, in this order, based on (**b**) the water sound speed profile. (**c**) Matched–filtered outputs of acoustic pressure and horizontal and vertical components of particle velocity scaled by acoustic impedance ρc, where *ρ* is water density and c is water sound speed.

In this section, we present simulations that were performed to verify the proposed method. An acoustic sensor was assumed to measure horizontal and vertical particle velocities in a 2D coordinate as well as acoustic pressure. The water depth of the simulation environment was 100 m, and a sound source transmitting a 200 ms-long linear frequency modulated (LFM) pulse with a bandwidth of 0.5–2 kHz was assumed to be positioned at a depth of 40 m. The receiver was assumed to be located at a depth of 80 m, and the range between the source and receiver was 700 m. A sandy sediment with a sound speed of 1814 m/s, density of 2.15 kg/$m^{3}$, and attenuation of 0.88 dB/λ was assumed to be a multipath-dominant waveguide. The underwater acoustic channel response for the given source–receiver geometry was predicted by BELLHOP [24], a ray-based propagation model, and convolved with the representation of the LFM pulse to simulate the received acoustic pressure signal. Then, isotropic white Gaussian noise was added (signal-to-noise ratio of 10 dB). The horizontal and vertical particle velocities were obtained by time integration of the pressure gradient and calculated via finite difference approximation. The pressure signals at two points were spaced 24 cm apart horizontally and vertically, which corresponds to one-fifth of the wavelength of the center frequency. Figure 2a shows the eigenray tracing output for six arrivals as follows: direct (D), bottom (B), sea surface (S), surface–bottom (S–B), bottom–surface (B–S), and bottom–surface–bottom (B–S–B) paths, in this order. These arrivals were calculated using the BELLHOP propagation model based on the sound speed profile shown in Figure 2b. Figure 2c shows the matched–filtered outputs of the horizontal and vertical components of particle velocity (scaled by acoustic impedance in water) and a comparison with that of acoustic pressure at the central point between two adjacent receivers.

**Figure 3:**
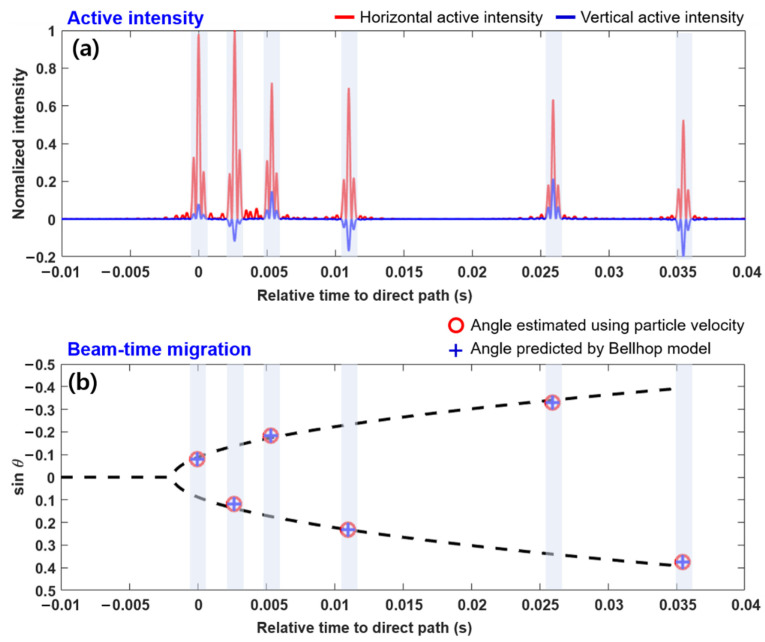
(**a**) Horizontal and vertical active intensity envelopes calculated from the simulated particle velocities for given simulation scenarios and (**b**) beam-time migration estimated for the multipaths. The vertical axis of (**b**) represents the beam angle (s=sinθ), where a positive value denotes an upgoing wave. The dashed line represents a beam-time migration curve obtained using the best fit value of χ, which is −2.09.

Figure 3a shows the horizontal and vertical components of active intensity obtained from Equation (7) by using the channel impulse responses shown in Figure 2c. The sign of the vertical active intensities corresponding to the upgoing wave after reflection from the bottom was negative. The arrival angle of each multipath could be easily estimated by calculating the arctangent of the intensity ratio of the peaks of the horizontal and vertical active intensities of each multipath. Figure 3b shows the beam-time migration results for the simulation scenario. The estimated beam angle structure of multipaths with time was in good agreement with that of the propagation model outputs.

Parameter χ was obtained by finding a gradient of the linear equation corresponding to Equations (2) and (3) that best fits the estimated multipath structure separated in beam angle and travel time. The best-fit value of χ was estimated to be −2.09, and the corresponding beam-time migration curve is indicated by the dashed line in Figure 3b. The horizontal range between the source and receiver was estimated using Equation (5) with β=0.986. For a source located 700 m from the receiver, the range was estimated to be 709 m with a range error of ~1.2%.

**Figure 4:**
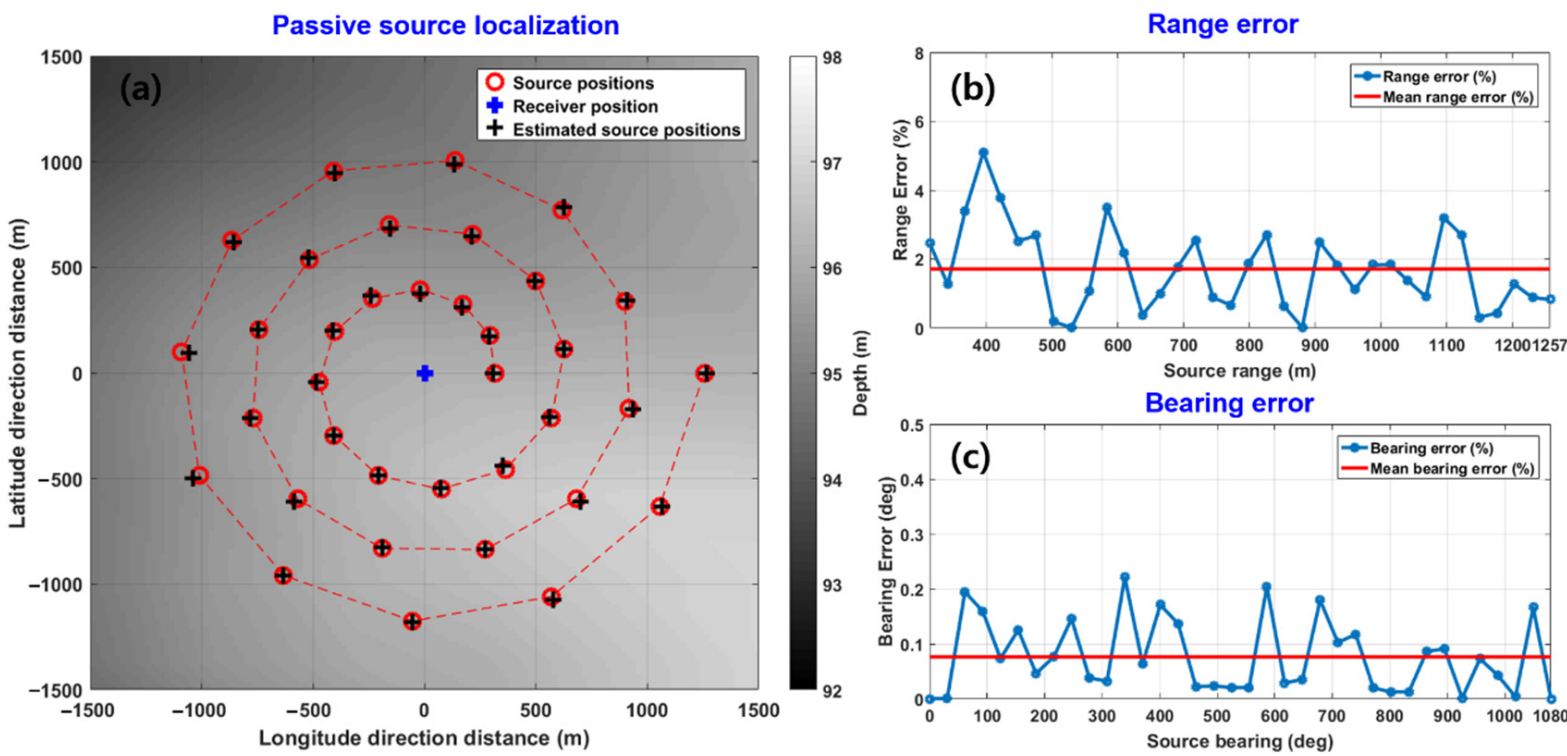
(**a**) Simulation results of passive source localization using the three components of acoustic intensity. The red circles indicate 36 source positions distributed in a spiral pattern and the cross symbols indicate the estimated source positions. (**b**) Range errors and (**c**) bearing angle errors for source localization estimates according to the source–receiver range.

Given that acoustic intensity has x, y, and z components, it can be extended to source localization in a 3D coordinate system. In this case, the azimuth angle to the source position can be estimated as the arctangent of the ratio of the x and y components of active intensity. The azimuth angle of source position *ϕ* can be estimated by

(8) $$\phi =tan^{-1}\left(\frac{I_{x}\left(t\right)}{I_{y}\left(t\right)}\right),$$

where $I_{x}$ and $I_{y}$ are the x and y components of active intensity, respectively. Figure 4 shows an example of the simulation results for 3D source localization. A total of 36 acoustic sources with different ranges and directions were assumed to be distributed in a spiral pattern in an area of 3 km × 3 km. A receiver that can measure the acoustic intensity vector was assumed to be positioned in the center of the area, as shown in Figure 4a. The sound speed profile shown in Figure 2b was used in the simulation, and the seabed was assumed to consist of coarse sandy sediment with a sound speed of 1814 m/s, density of 2.15 kg/$m^{3}$, and attenuation of 0.88 dB/λ, which were the same as those used in the previous simulation. The water depth was between 92 and 98 m. The simulation results showed that passive source localization using acoustic particle velocity worked well at various source positions (Figure 4a), with a mean range error of 1.7% (Figure 4b) and a bearing angle error of less than 0.1% (Figure 4c) for the given simulation scenario.

## 4. Experimental Description

**Figure 5:**
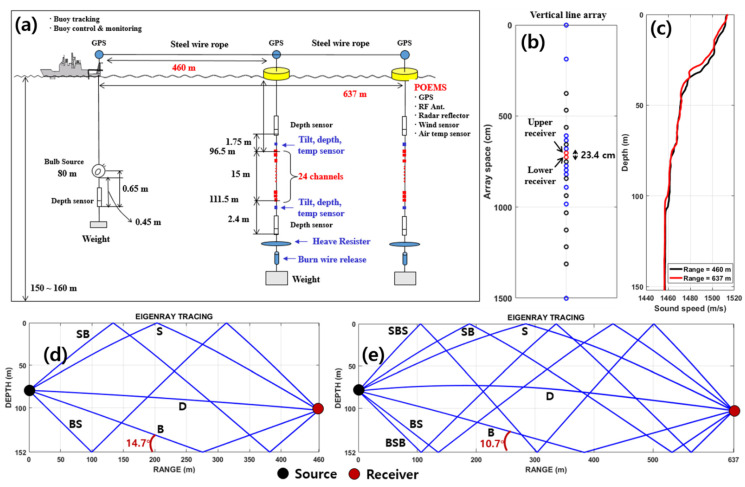
(**a**) Experimental geometry. (**b**) Layout of POEMS showing the spacing between 24 hydrophones. Two hydrophones in the middle of the POEMS and 23.4 cm apart were selected, of which the midpoint was 103.7 m-deep. (**c**) Sound speed profiles measured by XBT at 0218 and 0342 UTC. Eigenray outputs for source–receiver ranges of (**d**) 460 m and (**e**) 637 m based on the sound speed profiles. Grazing angles of B paths are 14.7° and 10.7° for 460-m and 637-m ranges, respectively; they are smaller than the estimated critical angle (26.8°) and therefore produce strong B paths.

Source localization was estimated using the particle velocity estimated by a finite difference approximation of the pressure signals measured by a vertical line array to verify the feasibility of the proposed method. The data were acquired from an acoustic experiment conducted on 13 July 2009, in shallow water located at 37°33.2′ N, 129°13.7′ E, which is approximately 10 km off the eastern coast of Korea. The water depth at the site varied within the range of 150–160 m (Figure 5a). As a sound source, a 500-W incandescent light bulb with a height of 12 cm and a width of 11 cm was deployed from the stern side of R/V Sunjin and broken at a depth of 80 m using a messenger. Bulb signals were acquired by a vertical line array called the Portable Ocean Environment System (POEMS) (Figure 5a), which is a 15 m-long nested array consisting of four subarrays with a total of 24 hydrophones. POEMS was moored such that the center of the array was at a depth of 103.7 m. POEMS is composed of four subarrays with hydrophone spacings of 187.5, 93.8, 46.9, and 23.4 cm. In this study, the two pressure signals received at two adjacent hydrophones with 23.4 cm spacing were used to estimate particle velocity, which was utilized for the passive source range estimate (Figure 5b). Given that the bulb signal has the characteristics of a broadband energy spectrum [25], the received bulb signals were bandpass-filtered with a bandwidth of 200 Hz and a center frequency of 800 Hz.

The lightbulb source and POEMS were placed approximately 460 and 637 m apart at 0218 and 0342 UTC, respectively, and those distances were kept while adjusting the length by connecting a steel wire rope between R/V Sunjin and POEMS during the measurements. The sound speed profiles, measured by an expendable bathythermograph (XBT), tended to decrease slightly with depth in both cases, as shown in Figure 5c. The surficial sediment collected by the piston sampler consisted of very fine sand with a mean grain size of 4.0 *ϕ*, and thus, the sound speed of the surficial sediment was estimated to be about 1633 m/s using the empirical formula given in Jackson and Richardson [26]. The wind speed during the acoustic measurements was less than 4 m/s and the sea state was kept calm. Figure 5d,e show the eigenray tracing outputs at a source depth of 80 m and receiver depth of 103.7 m for the source–receiver ranges of 460 and 637 m, respectively. The critical grazing angle was estimated to be 26.8° based on surficial sediment and water sound speeds above the seafloor. Therefore, the bottom paths for the nominal source–receiver ranges of 460 and 637 m, in which the grazing angles were 14.7° and 10.7°, respectively, can be considered to be totally reflected into the water medium, thus producing strong multipaths.

## 5. Results of Source Localization

Particle velocity can be obtained using the pressure gradient at two adjacent points, as explained in Section 2.2. In our case, particle velocity was estimated from the finite difference between the acoustic pressures measured by two hydrophones 23.4 cm apart, which corresponds to one-eighth of the wavelength at the center frequency (800 Hz) (Figure 5b). Since POEMS is the vertical line array, only the vertical component of the particle velocity was estimated. The mean acoustic pressure $p_{M}$ of the two hydrophones can be assumed as the acoustic pressure at the midpoint between two hydrophones [18,19,20,21,22,23], which is given by

(9) $$p_{M}\left(t\right)=\frac{p_{1}\left(t\right)+p_{2}\left(t\right)}{2}.$$

In our case, $p_{M}$ represents the acoustic pressure at a depth of about 103.7 m.

**Figure 6:**
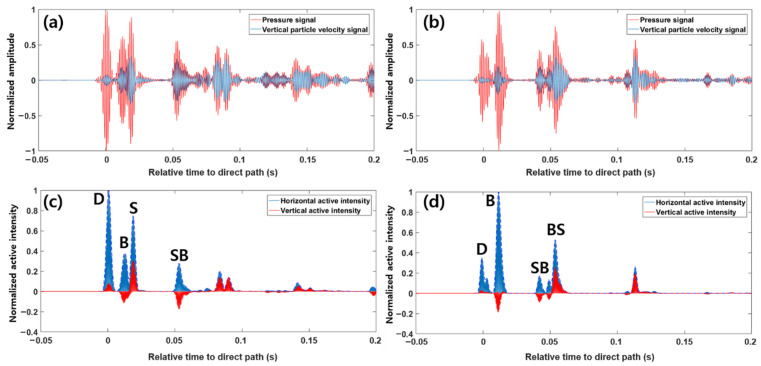
Bandpass-filtered pressure signals and estimated vertical particle velocities for source–receiver ranges of (**a**) 460 m and (**b**) 637 m; (**c,d**) show the estimated horizontal and vertical active intensities for the two ranges. The small peak between the D and B paths in (**d**) is the bubble pulse due to bubble oscillation, of which a detailed description is given later in this section.

Figure 6a,b show the bandpass-filtered pressure signals and their comparisons with the estimated vertical components of particle velocity for source–receiver ranges of 460 and 637 m, respectively. In both cases, the arrival structure showed characteristics with a distinct multipath and sparse delay spread due to the strong bottom-reflected paths. Then, the vertical active intensity was estimated from the real value of $I_{c}$ obtained with Equation (7). Assuming that the total active intensity is plane-wave intensity, the horizontal component of active intensity ${\tilde{I}}_{r}$ can be approximated from the vertical component of active intensity $I_{z}$ and the total active intensity, as follows [23]:

(10) $${\tilde{I}}_{r}=\sqrt{{\left(\frac{{\left|p\right|}^{2}}{\rho c}\right)}^{2}-I_{z}^{2}}.$$

There is a limitation in that the magnitude of the total active intensity may be underestimated in regions of strong destructive interference, which often occurs when multiple upward and downward propagation waves are overlapped in time [23].

Figure 6c,d show the estimated vertical active intensity and the horizontal active intensity obtained by Equations (7) and (10) for source–receiver ranges of 460 and 637 m, respectively. In these cases, although it was a multipath-dominant environment, Equation (10) was used to estimate the horizontal active intensity because the paths were well separated in time. However, in the case of the source–receiver range of 637 m, the S path immediately followed the B path with a time difference of ~1.5 ms, but it was simulated to be dominated by the B path due to the relatively large transmission loss caused by refraction near the sea surface.

The beam-time migrations were derived from the arctangent to the ratio of the horizontal and vertical components of active intensity with respect to arrival time with grazing angles (i.e., θ < 30°), which corresponded to the arrivals interacting with the ocean boundaries less than twice. Song and Cho [5] showed that it was reliable to compare the beam-time migration curve predicted using β=1 and the measured values for low-order modes with arrival angles less than about 23.6° (|s|<0.4). However, multipaths, especially in relatively short source–receiver ranges, often do not meet this condition, and this is also the case for our data. To take into account that the accuracy decreases as the arrival angle increases, the weighted least-squares (WLS) approach using weight factors corresponding to 1−|sinθ| was applied for multipaths with an arrival angle less than 30°.

**Figure 7:**
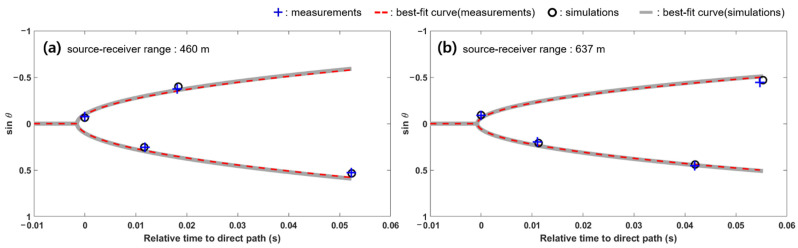
Estimated beam-time migrations and the best-fit elliptic curve solutions estimated using the measurement and simulation data for source–receiver ranges of (**a**) 460 m and (**b**) 637 m.

Figure 7a,b show the estimated results of beam-time migration (blue crosses) and the comparison with the best-fit beam-time migration curve (red dashed line) for source–receiver ranges of 460 and 637 m, respectively. The best estimates of χ were −3.21 and −2.29 for 460 and 637 m source–receiver ranges, respectively. The horizontal ranges between the source and receiver were estimated to be 460.5 and 644.2 m for 460 and 637 m ranges with β=1.016, which corresponded to range errors of 0.1% and 1.1%, respectively.

In this paper, because only the acoustic intensity of the vertical component was measured and used for estimation of the source–receiver range, it is necessary to confirm the reliability of the range estimation. For this reason, the simulation process was performed under the same environmental conditions as the sea experiment (see Figure 5). The channel impulse response at two vertically adjacent hydrophones was predicted by the BELLHOP acoustic propagation model as described in Section 3, and it was convolved with the reference bulb pulse to simulate the received acoustic pressure signal. Then, the source–receiver range was estimated from the simulated data using the array invariant method. Black circles and thick gray lines in Figure 7a,b represent the estimated beam-time migrations and the best-fit beam-time migration curves for source–receiver ranges of 460 and 637 m, respectively. The horizontal range between the source and receiver were estimated to be 457.7 and 631.7 m for 460 and 637 m ranges, which corresponded to range errors of 0.5% and 0.8%, respectively. The simulated results show that it is possible to apply the array invariant method after estimating the horizontal component from vertical acoustic intensity. However, as mentioned in Section 5, meticulous care is required when estimating the horizontal acoustic intensity using Equation (10) because there are some limitations.

The source localization results obtained using the acoustic intensity were compared with those obtained by the vertical line array consisting of pressure hydrophones. POEMS, which was used during the acoustic sea experiment, is a 15-m long nested array consisting of four subarrays. We used the second subarray consisting of 11 elements with a spacing of 93.8 cm (indicated by black circles in Figure 5b); thus, the design frequency was 800 Hz. The second subarray was 9.38 m long, and the center of the array was located at 105 m, which was approximately 1 m below the center of the two hydrophones used to estimate the particle velocity.

**Figure 8:**
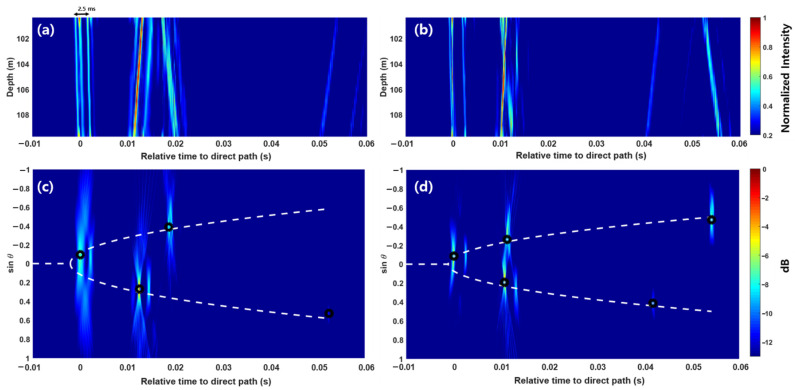
Channel impulse responses (CIR) measured at the vertical line array with 11 elements spanning a 9.38 m aperture for source–receiver ranges of (**a**) 460 m and (**b**) 637 m. Estimated beam-time migration (black circle) and the best-fit elliptic curve solution (white dashed line) for source–receiver ranges of (**c**) 460 m and (**d**) 637 m. The time difference between the first and third peaks in the first arrival group in (**a**) corresponds to the time difference between the first and second positive peaks of bubble pulses.

Figure 8a,b show the arrival structures of multipaths for ranges of 460 and 637 m, respectively, as a function of depth and time. Given that the source signal was generated by the underwater implosion of the light bulb, the waveform appeared in the form of a sequence of impulsive-shaped signals arising from the bubble oscillation. The waveform usually lasts for tens of milliseconds with an exponential decay until the pressure in the bubble and the hydrostatic pressure are balanced [25,27]. The period of the bubble pressure oscillation depends on the volume and implosion depth of the light bulb. In other words, the smaller the bulb volume and the deeper the implosion depth are, the shorter the bubble pulse period is [25]. In our case, the time difference between the first and second positive peaks of bubble pulses was 2.5 ms, which was consistent with the predicted value obtained by the semiempirical formula based on the modified Rayleigh–Willis formula presented in Porter and Bucker [25]. As clearly shown in Figure 8a, the first three dominant peaks in the first arrival group corresponded to the first positive, first negative, and second positive peaks of the bubble pulse sequence for the direct path in this order.

Figure 8c,d show the conventional beamforming results expressed as a function of s and time for the ranges of 460 and 637 m, respectively. On the basis of the beamforming results, the process to identify the representative point of each multipath was performed by following the method described in References 7–10, such as finding the peak exceeding the threshold, clustering the peaks, and identifying the maximum peak in each cluster. The χ values were then estimated to be −3.19 and −2.30 for the ranges of 460 and 637 m, respectively, by using the same method used above, namely, the WLS method with a weighting factor of 1−|sinθ|. Afterward, the source–receiver ranges for 460 and 637 m were determined to be 463.1 and 642.6 m, with range errors of 0.7% and 0.9%, respectively.

A comparison of the performance of the source–receiver range estimates using acoustic intensity and the beamforming data is presented in Table 1. Given that the range error may have been included in the source–receiver range measured during the experiment, it is difficult to ascertain which of the two methods had better performance through the performance comparison shown in Table 1. However, both methods exhibited good performance. In particular, the proposed source localization method using acoustic intensity showed a performance that is comparable to that of using the vertical array with 9.38 m length, even though only two hydrophones located at a short distance of about 23 cm were used.

## 6. Summary and Conclusions

The array invariant technique has been successfully applied to achieve robust source–range estimation in shallow water environments. However, it requires beamforming using a relatively long receiver array. In this study, the array invariant method using acoustic intensity was used for passive source localization. Given that acoustic intensity is a 3D vector quantity, source localization estimation using acoustic intensity can be more efficient in estimating beam-time migration than the use of array receivers that require beamforming techniques. Our simulation results for 36 acoustic source positions distributed in a spiral pattern on a plane demonstrated that satisfactory performance can be obtained with the three directional components of acoustic intensity.

The bulb signals acquired during the acoustic experiment conducted in shallow water off the eastern coast of Korea in July 2009 were used to verify the feasibility of the proposed method. A 500-W bulb was used as a sound source in the experiment, which was broken at a depth of 80 m. Finite difference approximation was used to estimate the vertical component of particle velocity because bulb signals were acquired using a vertical line array. Then, the vertical and horizontal active intensities were estimated and used in the array invariant algorithm for passive source localization. The array invariant parameter χ was estimated using the WLS method with the weight vector corresponding to 1−|s| for multipaths with an arrival angle of less than 30°. The horizontal source–receiver ranges were then estimated with Equation (5). Afterward, the results were compared with those obtained from beamforming of the acoustic signals acquired by the vertical line array. The comparison results showed that the proposed source localization method using acoustic intensity exhibited a performance comparable to that of using the relatively long vertical array. Our approach that uses acoustic intensity assumes that the acoustic signal corresponding to each multipath has purely active intensity. However, in a shallow water acoustic field, the inference among multipath arrivals activates reactive and active intensities, making it difficult to estimate the directions of arrivals.

In this study, particle velocity was obtained from two adjacent hydrophones via finite difference approximation. Using a vector sensor or a vector sensor array that can directly measure particle velocity or acoustic intensity vector may be more effective in estimating source localization in multipath-dominant shallow-water environments and may be applied extensively to underwater target tracking. However, although several mechanical and signal processing issues are beyond the scope of this study, they still need to be considered when using vector sensors; these issues include the effect of reactive intensity on vector sensor beamforming [28], calibration of the sensor’s reference frame (pitch, roll, and heading), and noise suppression (including nonacoustic noise contamination) [16,17,18]. Therefore, further studies are needed to verify the usefulness of vector sensors in source localization or target tracking in underwater environments.

## Figures and Tables

**Table 1:** Source–receiver ranges estimated based on beam-time migrations obtained using particle velocity and vertical line array.

Source Range	Acoustic Intensity (23.4 cm Aperture)		Vertical Line Array (9.38 m Aperture)	
	Estimated Range	Relative Error	Estimated Range	Relative Error
~460 m	460.5 m	0.1%	463.1 m	0.7%
~637 m	644.2 m	1.1%	642.6 m	0.9%

## Data Availability

Not applicable.
